# Combination gene therapy for HIV using a conditional suicidal gene with CCR5 knockout

**DOI:** 10.1186/s12985-021-01501-7

**Published:** 2021-01-30

**Authors:** Tugba Mehmetoglu-Gurbuz, Rose Yeh, Himanshu Garg, Anjali Joshi

**Affiliations:** 1 Department of Molecular and Translational Medicine, Center of Emphasis in Infectious Diseases, Texas Tech University Health Sciences Center, 5001 El Paso Dr, El Paso, TX 79905 USA; 2 Paul L Foster School of Medicine, Texas Tech University Health Sciences Center, El Paso, TX USA

**Keywords:** HIV, Gene therapy, HIV cure, CCR5, CXCR4, TK-SR39, Conditional, Cytotoxic, Ganciclovir, CRISPR

## Abstract

**Background:**

Gene therapy approaches using hematopoietic stem cells to generate an HIV resistant immune system have been shown to be successful. The deletion of HIV co-receptor CCR5 remains a viable strategy although co-receptor switching to CXCR4 remains a major pitfall. To overcome this, we designed a dual gene therapy strategy that incorporates a conditional suicide gene and CCR5 knockout (KO) to overcome the limitations of CCR5 KO alone.

**Methods:**

A two-vector system was designed that included an integrating lentiviral vector that expresses a HIV Tat dependent Thymidine Kinase mutant SR39 (TK-SR39) and GFP reporter gene. The second non-integrating lentiviral (NIL) vector expresses a CCR5gRNA-CRISPR/Cas9 cassette and HIV Tat protein.

**Results:**

Transduction of cells sequentially with the integrating followed by the NIL vector allows for insertion of the conditional suicide gene, KO of CCR5 and transient expression of GFP to enrich the modified cells. We used this strategy to modify TZM cells and generate a cell line that was resistant to CCR5 tropic viruses while permitting infection of CXCR4 tropic viruses which could be controlled via treatment with Ganciclovir.

**Conclusions:**

Our study demonstrates proof of principle that a combination gene therapy for HIV is a viable strategy and can overcome the limitation of editing CCR5 gene alone.

## Background

Recent advances in gene therapy and stem cell manipulation have renewed interest in developing a cure for HIV infection. The studies with the Berlin patient, wherein HIV co-receptor deficient cells from a CCR5delta32 homozygous individual were used to regenerate an HIV resistant immune system have demonstrated the viability of this approach [1, 2]. More recently, a similar approach was used on the London patient with apparent success [3]. Nevertheless, the study by Kordelas et al. showed that this approach has limitations as the virus can switch co-receptor usage to CXCR4 resulting in high levels of virus replication [4]. To overcome this, we explored the possibility of using a combination gene therapy that targets CCR5 along with a fall back approach of using a HIV-1 Tat dependent suicide gene.

The use of conditional cytotoxic gene, TK-SR39, and the potential of this approach to eliminate HIV infected cells has been previously studied by our group [5]. Previously, we have demonstrated that in cells expressing Tat dependent TK-SR39, HIV replication could be restricted by treatment with Ganciclovir [5]. This was true for both CXCR4 and CCR5 tropic viruses. However, this approach will require treatment with an FDA approved antiviral agent Ganciclovir, either continuously or when virus replication is observed. A strategy like CCR5 knockout (KO) is ideally suited to combine with suicidal gene therapy approach to achieve broader control of diverse HIV isolates. In fact, based on mathematical modeling, Pandit and de Boer proposed that targeting HIV entry alone via disruption of CCR5 will not be sufficient to reduce viral load to a level that will permit discontinuation of HAART. Furthermore, this study suggests that combination of CCR5 KO with a suicide gene would be a better strategy for anti-HIV gene therapy approaches [6].

The use of CCR5delta32 homozygous Hematopoietic Stem Cells (HSC) derived from an allogenic donor has an advantage of the cells being uniformly deficient in CCR5 expression. This was most likely the basis for the cure of the Berlin patient [1, 2]. This suggests that CD34 + HSC transplantation based therapies are most likely to be successful if all the transplanted cells are uniformly gene modified. In this regard, transduction efficiencies in CD34 + stem cells can be a limiting factor as increase in transduction often comes with a loss pluripotency [7]. Thus, development of gene therapy approaches with a selection marker to enrich modified cells need to be developed. Transient expression of GFP on genetically modified cells can be used as a feasible approach to achieve these goals.

Our study provides proof of principle for an anti-HIV gene therapy approach combining CCR5 KO with a conditional cytotoxic gene. This was achieved via a dual transduction strategy which also incorporated a selection marker (GFP) to allow enrichment of homogenous cell population with the desired gene modification. The approach utilized both an integrating lentiviral vector for stable integration of the TK-SR39 gene combined with a transient expression of CCR5gRNA-CRISPR/Cas9 and Tat via a non-integrating vector. This approach allowed for stable TK-SR39 integration, CCR5 gene knock out and transient GFP expression for cell enrichment. A stable cell line generated using this approach was resistant to infection with a CCR5 tropic HIV isolate. However, the cells were susceptible to CXCR4 tropic virus which could be restricted by treatment with Ganciclovir. To the best of our knowledge, this is the first study exploring a gene therapy approach that combines CCR5 KO with a Tat dependent suicide gene for HIV cure. Further studies in CD34 + stem cells and appropriate animal models will be needed to test the feasibility and further development of this strategy.

## Methods

### Cells, transfection and reagents

293T cells were maintained in DMEM supplemented with 10% Fetal Bovine Serum (FBS). Transfections were performed using the Turbofect reagent (ThermoFisher Scientific). TZM cells were obtained from NIH AIDS Reagent Program and cultured in DMEM supplemented with 10% FBS. TZM infections were performed in the presence of 20–40 µg/ml DEAE dextran (Sigma). TZM-TK-SR39 cells derived from TZM cells stably transduced with conditional Tat dependent TK-SR39 vector have been described previously [5]. The anti-herpes simplex virus drug Ganciclovir (GCV) was from Sigma.

### DNA constructs and cloning

CCR5gRNA-CRISPR/Cas9 was an all in one lentiviral vector expressing CCR5gRNA CRISPR/Cas9 and codon optimized HIV Tat. The vector was constructed by GenScript using gene synthesis technology (Additional file 1: Figure S1). Two separate vectors containing different sgRNA sequences against CCR5 and a control vector with a scrambled sgRNA sequence were synthesized. The CCR5gRNA sequence used in the study was 5′-TCAGTTTACACCCGATCCAC-3′. Vector pNL-GFP-RRESA-TK-SR39 is a lentiviral transfer vector that expresses TK-SR39 and GFP under the control of HIV Tat and has been described previously [5]. The vector is referred to as TK-SR39 for ease in this manuscript. Helper constructs pHP-dl-N/A and VSV-G were obtained from the NIH AIDS Reagent Program. The non-integrating lentiviral packaging vector LENTI-Smart NIL was from InvivoGen. The full length HIV proviral clones pNL-Lai and pNL-YU2 have been previously described [8].

### Virus stock preparation and concentration

For preparation of TK-SR39 virus stocks, 293T cells were transfected with the packaging DNA construct (TK-SR39 vector), helper DNA (pHP-dl-N/A) and VSV-G plasmid. For preparation of CCR5 gRNA-CRISPR/Cas9 Tat-NIL virus stocks, cells were transfected with respective DNA cloned into the lentiviral vector, LENTI-Smart NIL packaging vector and VSV-G DNA. Virus stocks were harvested 48 h post transfection and either used directly for infection or after concentration using the Viva Spin Columns (Sartorius) or ultracentrifugation at 20,000×*g*/90 min/4 °C. For preparation of Lai and YU-2 virus stocks, 293T cells were transfected with the respective full length infectious molecular clones. Supernatants were harvested 48 h post transfection and virus titers determined via infection of TZM cells.

### Titration of virus stocks and transductions

The TK-SR39 lentivirus particles were titrated in Jurkat-Tat cells while the Tat NIL particles were titrated in TZM-TK-SR39 cells. Briefly, cells were infected with twofold dilutions of virus stocks starting with a maximum of 100 µl in the presence of polybrene at 10 µg/ml (Jurkat-Tat) or DEAE dextran (TZM-TK-SR39) at 20–40 µg/ml and GFP expression detected 48 h post transduction.

### CCR5 knockout and western blotting

CCR5 knock out was determined by measuring cell surface CCR5 expression via flow cytometry and at the genetic level by PCR followed by T7 endonulcleaseI (T7EI) digestion. For cell surface CCR5 staining, the antibody CD195-PE was obtained from BD Biosciences and used as per the manufacturer’s recommendation. For genomic CCR5 disruption, the Alt-R Genome Editing Detection Kit was used (IDT) following the manufacturer’s protocol.

To determine expression of TK protein, cells lysates were run on 4–12% NuPAGE Bis–Tris gels (Invitrogen) and transferred onto PVDF membranes (Invitrogen). Blots were blocked with 5% non-fat milk in Tris Buffered Saline-Tween 20 (TBS-T), followed by incubation with primary anti-TK antibody (Santa Cruz Biotechnology) (1:1000) overnight at 4 °C. After 3 washes in TBS-T, the blots were incubated with horseradish-peroxidase conjugated secondary antibody (1:10,000) (Sigma). The blots were developed by enhanced chemiluminescence substrate using the Super Signal West Femto Maximum Sensitivity Substrate (Pierce) and images acquired using the SyngeneG gel documentation system.

### Flow cytometry and cell sorting

For determination of CCR5 knock out or GFP expression after transduction with different lentivirus stocks, cells were harvested and run on a 10 color Gallios flow cytometer (Beckman Coulter). At least 20,000 events for each sample were acquired. Data was analyzed using FlowJo software (Tree Star). For sorting of the CCR5 negative cells, the TZM-TKSR39 cells were transduced with the CCR5gRNA-CRISPR/Cas9 and Tat expressing non-integrating lentiviral particles. Cells were monitored for loss of GFP expression and CCR5 knock out over a period of time. The GFP^−^ and CCR5^−^ cells were sorted using the MoFlo Astrios EQ, Cell Sorter (Beckman Coulter).

### Cytotoxicity assay

Cells seeded in 96 well plates were induced to express TK-SR39 gene via transduction with NL-Luc/VSV-G lentiviral particles and treated with various concentrations of GCV. Cell viability was determined 48 h later using the CellTiter-Glo luminescent cell viability assay (Promega) that determines the number of viable cells based on quantitation of ATP as an indicator of metabolically active cells. Plates were read for luciferase activity using the FLUOstar Omega plate reader (BMG Labtech) and data analyzed using the Omega Data analysis software.

### Virus replication assay

For multiple round replication studies, parental TZM, TZM-TK-SR39 or the H7 cells were infected with pre-determined amount of NL-Lai virus or NL-YU2 virus for 3–4 h at 37 °C. The cells were treated with 5 µg/ml of GCV immediately post infection. The cells were sub cultured as needed and culture supernatants harvested for determination of infectious virus. For infectious virus determination, TZM indicator cell line was infected with equal amounts of harvested culture supernatants. Luciferase activity was determined 24 h post infection using the BriteLite plus reporter gene assay system (PerkinElmer) and plates read on a luciferase plate reader (BMG Labtech).

### Data analysis

Most assays were conducted in triplicates and data represent mean ± SD of triplicate observations. Data was analyzed and statistical analysis performed using the student’s t test in Microsoft Excel software. Flow cytometry data was analyzed using the FlowJo software (Tree Star). For fluorescent microscopy, images were acquired using the Nikon EclipseTi microscope and analyzed using the NIS Elements AR software.

## Results

### Combination anti-HIV gene therapy incorporating CCR5 KO with Tat dependent suicide gene

**Fig. 1:**
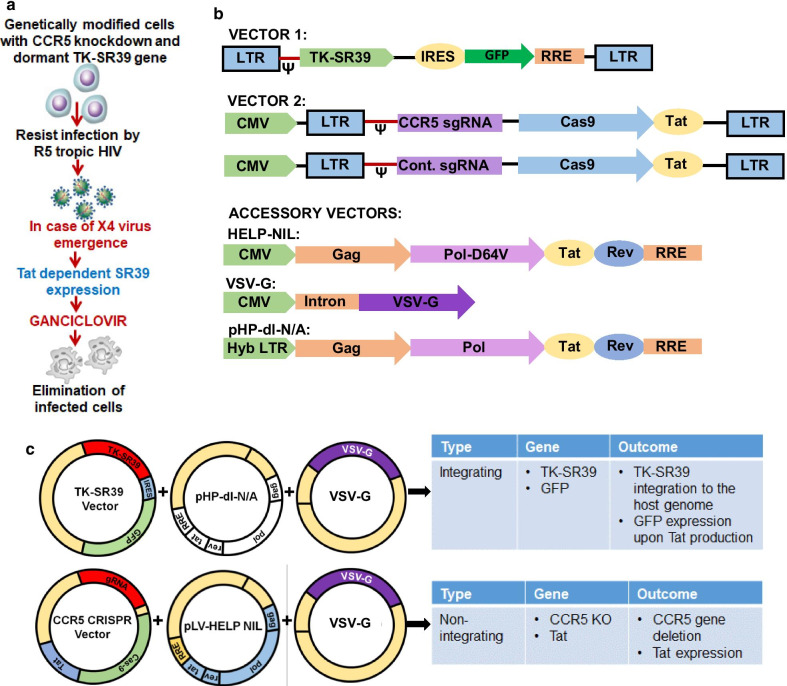
Strategy of combination gene therapy approach for HIV and plasmid maps. **a** Incorporating CCR5 KO along with conditional suicide gene to achieve HIV cure. Killing of HIV infected cells is achieved only in the presence of GCV providing an additional layer of safety for in vivo use. **b** Vector maps for the dual transduction strategy for HIV cure. Vector 1 incorporates TK-SR39 gene along with selection marker GFP downstream of an IRES. Vector 2 expresses the tat gene along with CCR5 gRNA-CRISPR/Cas9 cassette. **c** Strategy for packaging the vector 1 using an integrating lentivirus system and vector 2 using a non-integrating lentivirus resulting in permanent TK-SR39 integration, CCR5 KO and transient GFP expression for cell enrichment

Targeting the HIV co-receptor CCR5 via gene therapy has been the most extensively studied approach to limit HIV infection [9]. However, elimination of CCR5 expression via gene editing leaves the modified cells vulnerable to CXCR4 tropic viruses. To address this concern, we developed a combination gene therapy approach incorporating CCR5 KO along with a Tat dependent suicide gene TK-SR39. This approach would have two fold effects. Firstly, CCR5 KO would prevent entry of CCR5 tropic HIV isolates. Secondly, in the event of CXCR4 or dual tropic virus emergence, the Tat dependent TK-SR39 gene would be expressed leading to killing of infected cells in the presence of GCV (Fig. 1a). Furthermore, TK-SR39 cytotoxicity is dependent on the exposure of cells to GCV, adding a layer of safety for human gene therapy applications.

The combination gene therapy is delivered via a 2 step transduction approach (Fig. 1b, c). Vector 1 incorporates the TK-SR39 gene under the control of HIV LTR promoter and the GFP sequence downstream of an Internal Ribosomal Entry Sequence (IRES). Thus, TK-SR39 and GFP are expressed only in the presence of HIV Tat subsequent to HIV infection or ectopic Tat expression. As vector 1 is delivered via an integrating lentivirus, it is stably integrated into the host genome and capable of long term persistence in a dormant state as seen previously [5] (Fig. 1c). Vector 2 consisting of the CCR5gRNA-CRISPR/Cas9 and the tat gene, is delivered via a Non Integrating Lentivirus (NIL) as long term persistence these genes is not required (Fig. 1b). The transient expression via vector 2 over a period of 15–18 days provides the opportunity of CCR5 KO as well as transient GFP expression for cell selection (Fig. 1c). Thus, the two step gene therapy approach carries the potential to generate cells with CCR5 deletion along with TK-SR39 integration into the genome.

### Characterization of CCR5gRNA-CRISPR/Cas9 Tat vector for CCR5 KO, Tat and GFP expression

**Fig. 2:**
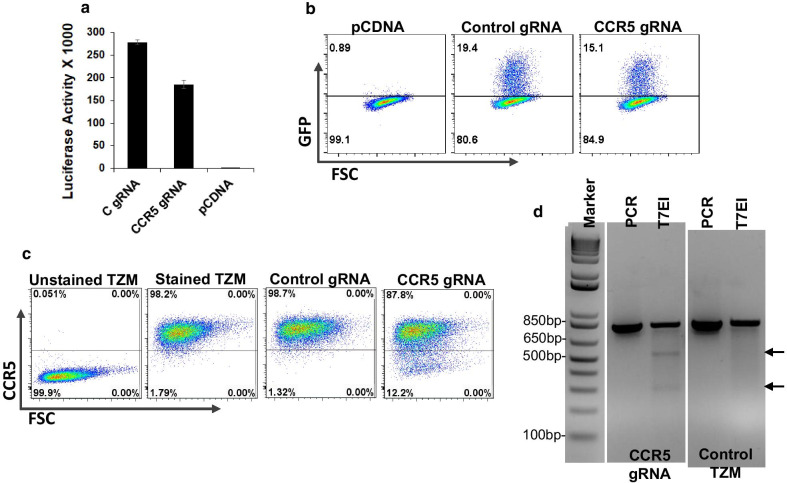
Characterization of Tat expression and CCR5 KO via vector 2. **a** TZM cells were transfected with CCR5gRNA-CRISPR/Cas9 Tat vector, Control-gRNA-CRISPR/Cas9 Tat vector or pCDNA3.1 empty vector. Luciferase activity was determined 48 h post transfection. **b** TZM-TKSR39 cells were transfected as in part A above. GFP expression was measured 48 h post transfection via flow cytometry. **c** TZM cells were transfected as in part A above. Six days post transfection, cells were analyzed for CCR5 expression by flow cytometry. **d** DNA was isolated from control TZM cells or TZM cells transfected with the CCR5gRNA-CRISPR/Cas9 Tat vector. Genetic disruption of the CCR5 locus was determined by PCR followed by T7EI digestion

The pNLGFPRRESA-TK-SR39 vector (Vector 1) used in this study has been described previously [5, 10]. However, the CCR5gRNA-CRISPR/Cas9 Tat vector (Vector 2) was developed for this study and was characterized for expression of Tat protein and CCR5 KO via transfection experiments. We utilized TZM cells as reporter cells to test these activities as the cells express CCR5 as well as a Tat dependent luciferase gene. As demonstrated in Fig. 2a, transfection of TZM cells with either CCR5gRNA or control gRNA vector resulted in increased luciferase activity confirming Tat expression. We further tested whether this Tat expression could induce GFP expression in cells stably integrated with the pNLGFPRRESA-TK-SR39 [5]. As demonstrated in Fig. 2b, transfection of TZM-TK-SR39 cells with control gRNA or CCR5gRNA expressing vector resulted in GFP expression as detected by flow cytometry. Finally, the vector was tested for its ability to knock out CCR5 in TZM cells by determining cell surface CCR5 expression by flow cytometry and genomic editing of the CCR5 locus by PCR. As demonstrated in Fig. 2c, TZM cells uniformly express CCR5 on their cell surface. Transfection with the CCR5gRNA vector resulted in loss of CCR5 expression while transfection with control gRNA vector resulted in no CCR5 KO. For genomic disruption of the CCR5 locus, we performed locus specific PCR followed by the T7EI digestion. The process resulted in digestion of the ~ 870 bp PCR fragment into two expected bands of ~ 533 and ~ 300 bp. As expected, no digestion of the PCR product was observed for control TZM cells. Overall, these findings indicate that our newly developed CCR5gRNA-CRISPR/Cas9 Tat construct is capable of Tat expression, CCR5 KO and induce GFP expression in appropriate cell types.

### Sequential transduction of TZM cells with TK-SR39 vector followed by CCR5gRNA-CRISPR/Cas9 Tat vector results in GFP expression and CCR5 knock out

**Fig. 3:**
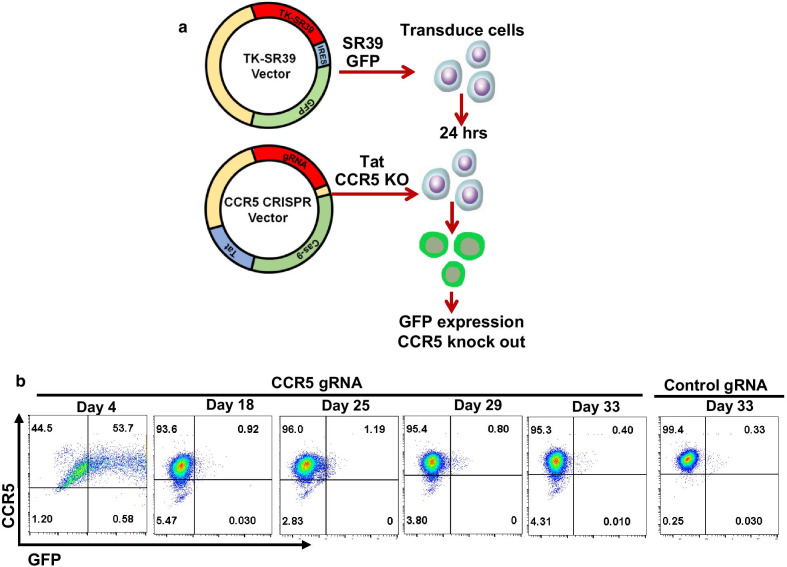
Dual transduction of TZM cells with TK-SR39 vector followed by CCR5gRNA-CRISPR/Cas9 Tat vector results in GFP expression and CCR5 KO. **a** Strategy for dual transduction of TZM cells with integrating lentiviral vector 1 to deliver Tat dependent TK-SR39 gene and with non-integrating lentiviral vector 2 to deliver Tat and CCR5gRNA-CRISPR/Cas9. GFP expression and CCR5 KO was determined over a period of time. **b** TZM cells were transduced with vector 1 followed by vector 2 as in part A. Down regulation of surface CCR5 expression was monitored over a period of time by flow cytometry

We next tested the feasibility of our dual transduction approach for HIV gene therapy in cell culture based system. TZM cells were first transduced with TK-SR39 construct packaged using an integrating lentiviral vector. Next day, the cells were transduced with the CCR5gRNA-CRISPR/Cas9 Tat construct packaged using a non-integrating lentiviral (NIL) vector (Fig. 3a). Cells were then monitored for GFP expression and CCR5 KO over a period of several days. As demonstrated in Fig. 3b, GFP expression was seen as early as day 4 post second transduction and was completely lost at ~ 15 days post transduction due to transient expression of Tat from the NIL vector 2. CCR5 down regulation was evident at day 18 and persisted till the end of the experiment on day 33 (Fig. 3b). Interestingly, while the efficiency of GFP expression was high and reached up to 90% in some experiments, the efficiency of CCR5 knock down was relatively low and did not exceed 6% in our dual transduction experiments. The abundance of GFP expression, yet poor CCR5 knock out reflects on the low efficiency of CRISPR knock out [11] rather than inefficient transduction via vector 2. Overall, our data demonstrate that the dual transduction anti-HIV gene therapy approach is viable but will require optimization of CRISPR knock out efficiency and/or selection of stable cell clones with desired genetic repertoire for practical applications.

### Generation of a stable cell line expressing Tat dependent TK-SR39 and CCR5 KO

**Fig. 4:**
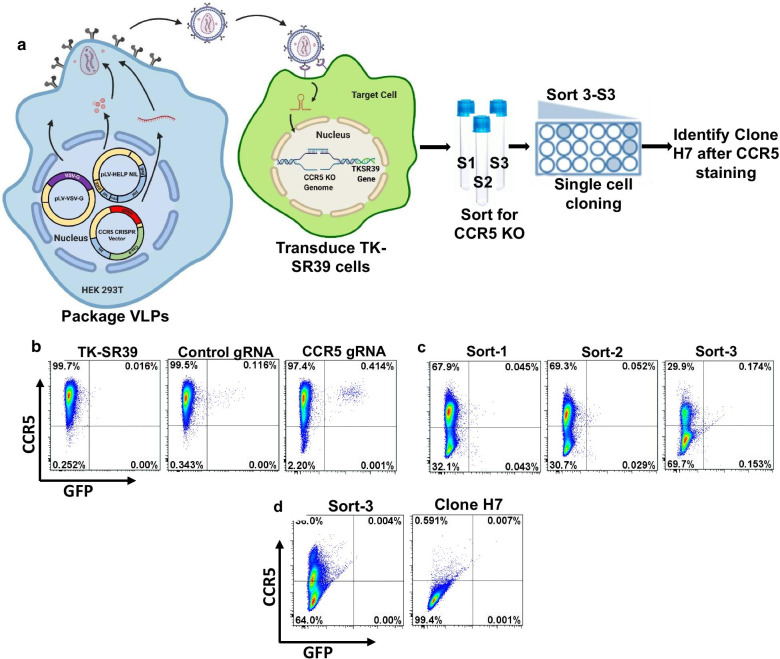
Generation of a stable cell clone with TK-SR39 integration and CCR5 KO. **a** 293T cells were transfected to generate CCR5gRNA CRISPR/Cas9 Tat NIL particles. TZM-TK-SR39 cells were transduced with the NIL lentivirus and the cells monitored for GFP expression and CCR5 down regulation. CCR5 negative cells were sorted 3 times to generate sort 1–3 (S1, S2, S3) followed by single cell cloning. **b** Flow cytometry analysis of (**b**). The TZM-TK-SR39 cells transduced with control gRNA packaged lentivirus or CCR5 gRNA lentivirus were stained for CCR5 expression prior to cell sorting. **c** The sorted cells (Sort 1–3) were assayed for surface CCR5 expression by flow cytometry. **d** Sort-3 cells were further subjected to single cell cloning and several potential clones screened for CCR5 downregulation. The selected clone H7 was stained for CCR5 and compared to the parental Sort-3 cells

As demonstrated in Fig. 3, the efficiency of CRISPR mediated CCR5 knock out was low in our hands using the dual transduction approach. We hence made use of our previously generated TZM-TK-SR39 cell line that incorporates a stably integrated TK-SR39 gene and has been characterized for inhibition of CCR5 and CXCR4 tropic HIV infections in the presence of GCV [5]. We transduced the TZM-TKSR39 cell line with the CCR5gRNA CRISPR/Cas9 Tat NIL lentivirus particles to generate a stable cell line expressing the TK-SR39 gene with CCR5 KO (Fig. 4a, b). After transduction, the CCR5 negative GFP negative cells were sorted via FACS three different times to generate bulk sorts 1, 2 and 3. As demonstrated in Fig. 4c, cells from sort 3 showed best CCR5 knock out when assayed for cell surface CCR5 expression. Hence, sort 3 was subjected to single cell cloning via limiting dilution in a 96-well plate. Several single cell clones were screened for cell surface CCR5 expression resulting in clone H7, which showed complete loss of cell surface CCR5 (Fig. 4d). Using this strategy, we were able to generate a stable cell clone that expresses the conditional cytotoxic gene TK-SR39 along with genetic deletion of CCR5.

### CCR5 KO H7 clone is resistant to CCR5 tropic HIV infection, expresses the TK gene in the presence of Tat and shows cytotoxicity in the presence of GCV

**Fig. 5:**
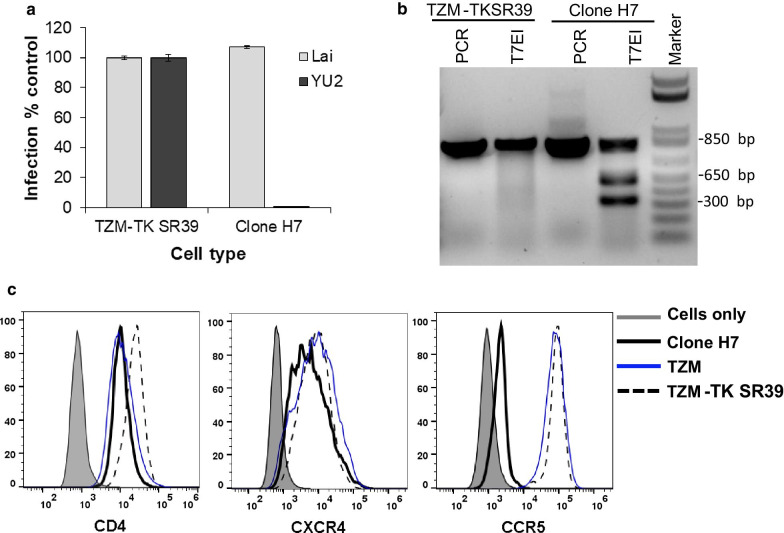
Characterization of H7 cell line for resistance to HIV infection by CCR5 tropic HIV **a** TZM-TKSR39 or H7 cells were infected with HIV reporter virus particles pseudotyped with Lai or YU-2 Envelope. Infection was determined 24 h post infection by measuring luciferase activity in the cultures. **b** DNA was isolated from TZM-TKSR39 cell line or H7 clone and site of CRISPR mediated CCR5 disruption amplified using specific primers. PCR product was digested using the T7EI and bands were resolved on an agarose gel. **c** TZM, TZM-TK-SR39 or H7 cells were stained for CD4, CXCR4 and CCR5 expression using specific antibodies and analyzed by flow cytometry

We next characterized the TZM-TKSR39 CCR5 KO cell clone (H7) for different characteristics like susceptibility to HIV infection, CCR5 KO while preserving CD4 and CXCR4 expression, TK gene expression and GCV mediated cytotoxicity in the presence of Tat. As depicted in Fig. 5a, the parental TZM-TK-SR39 cells were susceptible to infection with both CXCR4 tropic HIV-Lai and CCR5 tropic virus YU2. However, as expected, the H7 clone resisted infection with CCR5 tropic HIV YU-2 while still retaining susceptibly to CXCR4 tropic Lai. The lack of infection of H7 cells with CCR5 tropic HIV confirms CCR5 KO as they expressed the HIV receptor CD4 and the co-receptor CXCR4 comparable to WT TZM Cells (Fig. 5c). CCR5 KO in the H7 clone was also evident genetically via PCR followed by the T7EI treatment that resulted in digestion of the PCR product. However, for the parent TZM-TKSR39 cells, a single PCR amplified band was observed with no sub-products generated upon T7EI digestion (Fig. 5b).

**Fig. 6:**
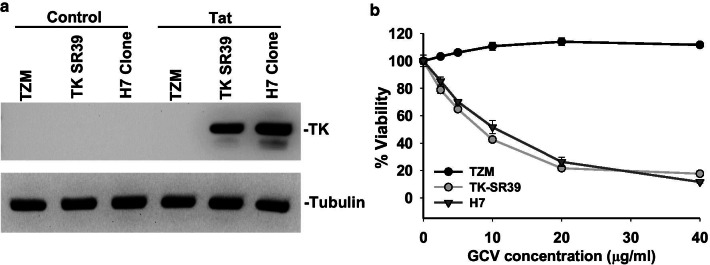
H7 cell line shows Tat dependent TK gene expression and cytotoxicity in the presence of GCV. **a** TZM, TK-SR39 or H7 cells were transduced with control or Tat expressing lentivirus particles. Cells were lysed 48 h post infection and TK gene expression determined by western blotting. **b** TZM, TK-SR39 or H7 cells were transduced as in part A above and treated with the indicated concentrations of GCV. Percent viability was determined 48 h post transduction using the Cell Titer Glo luciferase based assay

We next analyzed TK gene expression in the H7 clone in the presence of Tat. As demonstrated in Fig. 6a, in the absence of Tat, no TK gene expression was seen in the parental TK-SR39 cell line or its derivative H7 cell line. As expected, robust TK expression was seen in both TK-SR39 and H7 cell lines in the presence of Tat. TK expression mediated cytotoxicity was assessed by culturing the cells in the presence of different concentrations of GCV. As demonstrated in Fig. 6b, a dose dependent GCV mediated cytotoxic effect was seen only in the TK-SR39 and H7 cell line in the presence of Tat. TZM cells, on the other hand, showed no reduction in cell viability in the presence of GCV and Tat. Taken together, these findings demonstrate effective CCR5 knock out in the H7 cell line leading to lack of CCR5 HIV infection, tightly controlled TK expression only in the presence of Tat and cytotoxicity only in the presence of Tat and GCV.

### Dual modified H7 cells are resistant to CCR5 tropic virus infection and can resist CXCR4 tropic HIV replication in the presence of Ganciclovir

**Fig. 7:**
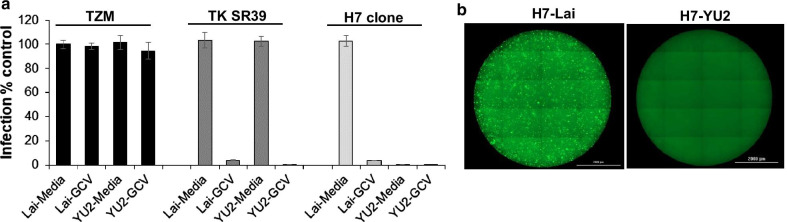
H7 cells can control replication of both R5 and X4 tropic HIV. **a** TZM, TK-SR39 or H7 cells were infected with replication competent Lai or YU-2 virus in the presence or absence of 5 µg/ml GCV. Culture supernatants were harvested at day 6 post infection and an equal volume of supernatant used to infect TZM cells. Percent infection in TZM cells was determined 24 h post infection. **b** H7 cells were infected with HIV Lai or YU-2 virus as in part A. Images of whole wells were acquired using the Cytation5 imager

The crux of an effective HIV gene therapy approach would be control replication of both CXCR4 and CCR5 tropic HIV isolates. We hence tested the ability of our H7 cell line to resist infection by CXCR4 and CCR5 tropic HIV isolates. As demonstrated in Fig. 7a, WT TZM cells efficiently replicated both CXCR4 and CCR5 tropic HIV isolates in the presence or absence of GCV. TZM-TKSR39 cells supported replication of both CXCR4 and CCR5 tropic HIV which could be inhibited via treatment with GCV consistent with our previous study [5]. The H7 clone on the other hand resisted CCR5 tropic virus infection due to a lack of CCR5 expression preventing virus entry (Fig. 7b). However, the cells were readily infected with CXCR4 tropic Lai as evident by GFP expression upon virus replication (Fig. 7b). While the H7 cells could be infected with CXCR4 tropic (Lai) virus, the replication of the virus could be inhibited with GCV treatment (Fig. 7a).

**Fig. 8:**
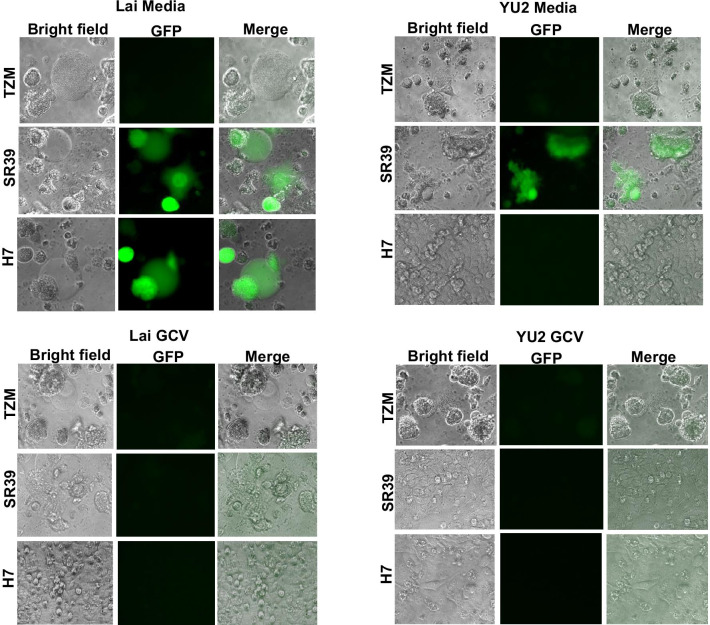
H7 cells resist R5 HIV infection and control X4 HIV via GCV treatment. (A) TZM, TK-SR39 or H7 cells were infected with replication competent Lai or YU-2 virus in the presence or absence of 5 µg/ml GCV. Cells were fixed at day 3 post infection and images acquired using the Nikon Eclipse Ti microscope

HIV replication in TZM cells is characterized by extensive syncytia formation. Control of virus replication results in cessation of this cytopathic effect. We hence looked at syncytia formation in TZM, TK-SR39 and H7 cells in the presence or absence of GCV when infected with Lai or YU-2 strains of HIV. As demonstrated in Fig. 8, infection of TZM-TK-SR39 and H7 cells with HIV-Lai resulted in extensive syncytia formation and GFP expression due to presence of Tat. Interestingly, addition of GCV abrogated syncytia formation and GFP expression in both TZM-TK-SR39 and H7 cells. As expected TZM cells showed no GFP expression but exhibited extensive syncytia formation, a phenomenon that was not affected by addition of GCV. With regards to YU-2 infection, TZM cells once again showed syncytia formation in the presence or absence of GCV. TZM-SR39 cells formed syncytia along with GFP expression that was inhibited in the presence of GCV. Interestingly, the H7 cell line showed no GFP expression or syncytia formation upon YU-2 infection with or without GCV establishing the resistance of this cell line to R5 tropic viruses. Thus, the H7 cell line resists infection by R5 tropic HIV due to absence of CCR5 co-receptor and can control infection of X4 tropic HIV via TK-SR39 expression and GCV treatment.

## Discussion

Advances in stem cell transplantation have revolutionized treatment of genetic disorders that were once conceived as incurable. The most notable amongst these have been for age related macular degeneration where an ultrathin scaffold of stem cells is used to replace the damaged cells resulting in vision restoration [12–14]. Other notable stem cell related genetic advances include treatment for Severe Combined Immunodeficiency (SCID), X-linked adrenoleukodystrophy, Wiskott-Aldrich Syndrome, diabetes, Parkinson’s diseases, third degree burns etc. [15–17].

HSC have also been used to cure the Berlin patient of HIV infection [1, 2]. He is the only person in long term viral remission after stem cell transplantation from a CCR5delta32 donor in 2007 and has been off anti-retroviral therapy since then. Similar strategy has been used for the treatment of the London patient who also received a stem cell transplantation from a CCR5delta32 donor [3] and was declared HIV free in 2019, eighteen months after cessation of anti-retroviral therapy. These two cases support the possible success of HIV resistant HSC transplantation in achieving an HIV cure. While considering CCR5 KO for HIV gene therapy, mono/bi allelic disruption of the CCR5 locus is an important point for consideration. In this context, some insights can be gained from studies with CCD5delta32 heterozygous individuals. Although these individuals are susceptible to HIV infection, they show delayed progression to AIDS possibly via lower CCR5 expression [18]. Our lab has extensively studied the role of CCR5 expression levels in HIV pathogenesis [19–25] and believe that a reduction in CCR5 levels in mono allelic KO may reduce CCR5 levels and provide a CCR5delta32 heterozygous like phenotype in patients resulting in reduced HIV pathogenesis. However, the goal of CCR5 KO should be bi allelic KO in order to achieve HIV resistance/cure as seen in the Berlin patient.

The use of CCR5 co-receptor deficient cells for achieving a HIV cure has been at the forefront of gene therapy approaches for HIV [9]. However, gene therapy mediated targeting of CCR5 alone raises the possibility of co-receptor switching by HIV to CXCR4 in the infected patients [26]. This fear was realized in the study by Kordelas et al. where CCR5delta32 stem cell transplantation in a HIV + patient led to co-receptor switching resulting in extremely high HIV titers [4]. The reason why co-receptor switching was seen in the study by Kordelas et al. and not the Berlin patient remains unclear. One possibility is the presence of CXCR4 tropic variants in the viral reservoir prior to gene therapy. Thus, it remains unknown if CCR5 KO gene therapy would drive co-receptor switching or there will be an outgrowth of pre-existing CXCR4 tropic variants. With co-receptor switching a possibility, the strategy of targeting CCR5 alone for an HIV cure remains incomplete. Nevertheless, several CCR5 targeting strategies including RNA interference, Zinc finger nucleases, TALENS, CRISPR/Cas9 have been tried in T cell lines, primary CD4 cells and HSC for CCR5 elimination in context of HIV disease [9, 27].

Besides targeting CCR5, other approaches to disrupt the HIV life cycle have been pursued like targeting the viral fusion process [28, 29], expression of antiviral genes like TRIM5alpha [30], RNAi mediated targeting of HIV genome [31], vectored delivery of broadly neutralizing antibodies [32–34] and use of CAR T cells to target the HIV gp120 [35, 36]. Dominant negative (DN) proteins like RevM10 [37], DN Gag [38] and Env genes [39] have also been pursued although they carry the caveat of virus evasion [40]. We previously demonstrated the efficacy of a combination of the DN Gag and Env viral proteins in inhibiting HIV in vitro [41]. Subsequently, we developed a conditional cytotoxic suicide gene therapy approach targeted to kill HIV infected cells in the presence of GCV [5]. In the current study, we developed a combination gene therapy approach combining CCR5 KO with the conditional suicide gene for targeting virus entry along with halting virus replication. This approach not only bears the advantage of being effective against a broader spectrum of CCR5 and CXCR4 tropic viruses, but resistance against SR39 mediated cell killing is unlikely. Moreover, control of cell killing via GCV administration adds another layer of safety making the approach attractive for further clinical development. GCV is a successful anti-HSV drug with an excellent safety profile. However, studies have suggested a bystander killing effect of GCV [42]. In our previous study [5] we conducted virus infection experiment where not all cells are infected at the same time, hence reproducing a co-culture/bystander cell scenario and found that increasing concentrations of GCV limits virus replication and concurrently increases the viability of uninfected cells. Thus, GCV treatment by itself does not seem to be a factor in enhancing bystander cell death in our system. Our data here provides proof of principle for a combination gene therapy approach administered via dual transduction to have efficacy against both CXCR4 and CCR5 tropic HIV. However, one obvious limitation was the limited efficacy of CCR5 KO in our hands using the CRISPR approach. Most of the CCR5 + cells lines used for HIV infection studies are ectopically engineered to express CCR5 and hence have multiple copies of CCR5 to achieve high CCR5 expression. However, TZM cells have been extensively used for both CCR5 and CXCR4 tropic HIV infection and hence were our choice of cells for the study. One of the major obstacles for translational use of CRISPR/Cas9 is that the efficiency of homology directed repair (HDR) mediated gene disruption/correction is limited and is affected by factors like cell type, state of cell division, accessibility to certain genomic sites, competition with the endogenous non-homologous end joining (NHEJ) etc. [43–45]. Efforts to increase CRISPR efficacy include options like better sgRNA complementarity [44], inhibiting the NHEJ pathway [44], chemical modifications [46–48], gRNA-donor DNA conjugation [49] etc. As gene targeting efficiency is improved with new generation CRISPR-Cas9 techniques with low off target effects, more gene therapy based clinical trials will appear in the pipeline in the next decade.

We do realize the limitation of the dual transduction strategy for our anti-HIV gene therapy approach. Combined with the low efficiency of CRISPR mediated CCR5 KO, especially in TZM cells, the translational utility of this approach remains limited. However, our approach does incorporate the added benefit of selection/enrichment of genetically modified cells via Tat mediated GFP expression. This selection strategy can overcome the apparent low efficiency of CRISPR/Cas9 mediated KO. A relatively new area of research gaining ground in terms of stem cell therapy is that of induced pluripotent stem cells (iPSCs). These are adult stem cells that can be genetically re-programmed into an embryonic stem cell like state and have the potential to differentiate into any cell type [50]. Thus, theoretically speaking, a single genetically modified cell would be sufficient to repopulate the entire immune system with CD4 helper cells that are resistant to HIV infection. The use of iPSCs was approved in 2013 by Japan’s Health Ministry for treating age related macular degeneration where iPSCs would be transplanted into the patient’s eyes [50]. Overall, our study provides evidence that a combination gene therapy approach for HIV incorporating transduction with two lentiviruses that have different targets is feasible. While TK-SR39 mediated cell killing in the presence of Tat and GCV is highly potent and specific, the CRISPR mediated editing of the CCR5 gene was limiting. The CRISPR KO field is rapidly evolving and new generation Cas9 proteins and enhanced delivery methods like nanoparticles are being developed. While we used a non-integrating vector we are confident that other methods of achieving more efficient CCR5 KO will be available soon and can be applied to our therapy.

## Conclusion

**Fig. 9:**
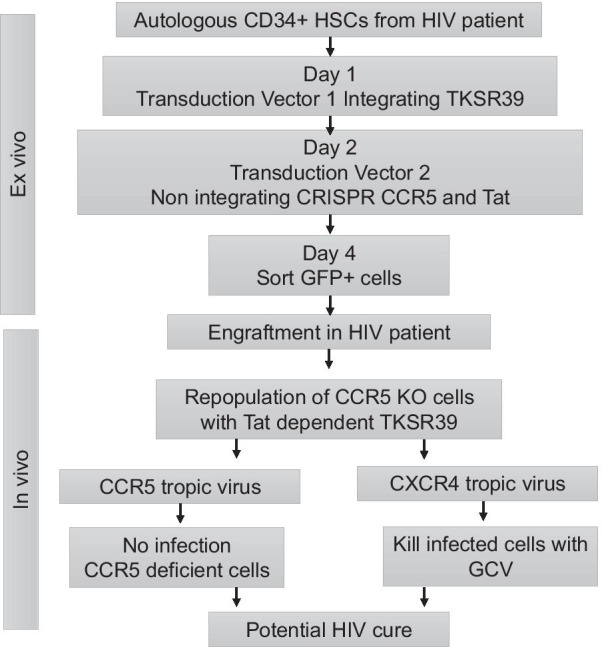
Overview of combination gene therapy for HIV combining CCR5 knock-out with a suicide gene

Our study was designed as a proof of principle study to demonstrate that a combination gene therapy for HIV combining CCR5 knock-out with a suicide gene is a better approach than a single target. Our approach compliments the CCR5 KO strategy by providing a fall back target in case of co-receptor switching in patients after modified HSC transplantation (Fig. 9). We acknowledge that there may be technical limitations like increasing CRISPR/Cas9 efficiency, selection of modified cells or expansion of stem cells from a single clone in our study. Future studies in relevant cell types are warranted to assess the therapeutic relevance of this approach.

## Supplementary Information

**Additional file 1: Figure S1.** Detailed vector maps of the Lenti CCR5gRNA CRISPR/Cas9 and TK-SR39 GFP vectors used in the study.

## Data Availability

The datasets used and analyzed during the current study are available from the corresponding author on reasonable request.

## Abbreviations

CCR5: C–C motif chemokine receptor 5
CXCR4: C–X–C chemokine receptor type 4
CRISPR: Clustered regularly interspaced short palindromic repeats
gRNA: Guide RNA
KO: Knock out
NIL vector: Non-integrating lentiviral vector
sgRNA: Single guide RNA
TK: Thymidine kinase
