# Supplementary figures and images for: Combination gene therapy for HIV using a conditional suicidal gene with CCR5 knockout
Source: Virol J. 2021 Jan 30;18:31. doi: 10.1186/s12985-021-01501-7 (PMC7847599; doi:10.1186/s12985-021-01501-7)

Supplementary Figure 1

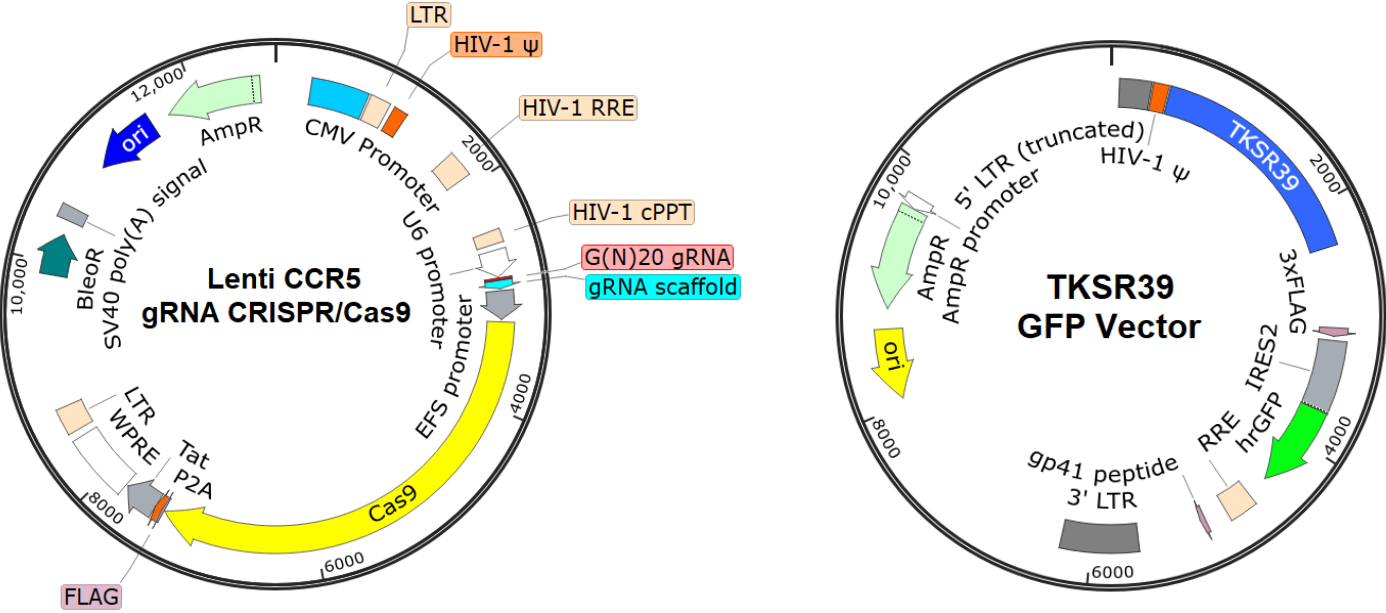

Supplement: Supplementary file 1 — Additional file 1: Figure S1. Detailed vector maps of the Lenti CCR5gRNA CRISPR/Cas9 and TK-SR39 GFP vectors used in the study. [file 12985_2021_1501_MOESM1_ESM.pdf]
